# Salivary Fistula as a Complication After the ORIF of a Mandibular Condylar Process Fracture: A Single-Centre Retrospective Study

**DOI:** 10.3390/jfb16090326

**Published:** 2025-09-04

**Authors:** Paulina Agier, Marcin Kozakiewicz, Piotr Szymor

**Affiliations:** 1 Multi-Speciality Dental Clinic, 106/116 Kośćiuszki Av., 90-442 Lodz, Poland; paulinaagier2402@gmail.com; 2 Department of Maxillofacial Surgery, Medical University of Lodz, 251 Pomorska St., 92-213 Lodz, Poland; piotr.szymor@umed.lodz.pl

**Keywords:** condyle, mandible, fracture, ORIF, surgery, complication, salivary fistula, risk factors

## Abstract

Surgical management of condylar process fractures is associated with postoperative complications, the most common being transient facial nerve palsy. Less frequent but noteworthy is the development of salivary fistulas, which, although rare, constitute a clinically relevant condition. This research aimed to investigate factors impacting salivary fistula formation and treatment in patients surgically treated for mandibular condylar process fracture. This study included 395 patients who underwent open rigid internal fixation (ORIF). Salivary fistula occurred in 5.8% of those treated. Multiple factors were assessed as potential contributors to post-operative fistula formation, but only gender demonstrated a statistically significant association as an independent risk factor (*p* < 0.05). The longer the surgical procedure, the sooner a fistula will appear in the postoperative follow-up period. Moderately elevated white blood cell and C-reactive protein levels were associated with faster resolution of salivary fistula. Treatment duration was longer for patients with a low body mass index. The most effective treatment method was disinfecting the fistula, applying a pressure dressing, and adhering to a tasteless diet (*p* < 0.05); both chemical cauterization and plastic surgery proved to be less effective. When a fistula occurs, it can be successfully resolved in a relatively short period of time (median 10 days); in most cases, conservative methods are sufficient. As this is a pioneering study, further research is necessary to validate the results.

## 1. Introduction

**Figure 1:**
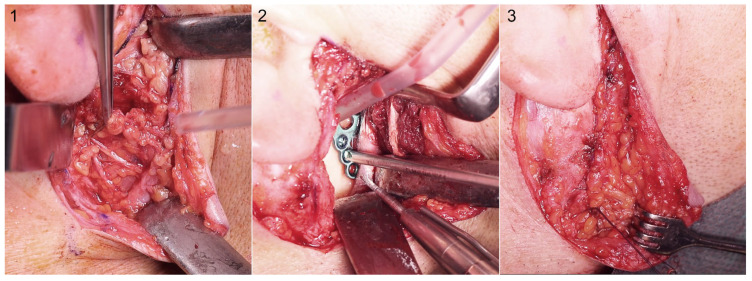
Open rigid internal fixation of the fracture in the condylar process of the mandible. (**1**): Typical transparotid approach in the identification phase of the facial nerve branches below the superficial lobe of the parotid gland. (**2**): Osteosynthesis by titanium plate. (**3**): Final watertight continuous interleave suture of parotid capsule together with preauricular fascia and superficial musculo-aponeurotic system.

Maxillofacial surgery is a traumatology-associated medical discipline. Consequently, many maxillofacial surgeons are routinely tasked with managing fractures and injuries resulting from various etiologies, including road traffic accidents, assaults, falls, sports-related contusions, and work-related incidents. The mandible has been identified as the most frequently fractured bone in the facial region [1,2]. The most common fracture of the mandible is to the body, with the second being to the condylar process [3,4]. Mandibular condylar process fractures present a clinical challenge owing to their small dimensions and exposure to high mechanical strains [5,6]. Accordingly, precise diagnosis and appropriate treatment planning are essential. Once the diagnosis is established, the choice of the most suitable therapeutic approach must be determined. The available options are closed, i.e., conservative treatment, or open rigid internal fixation (ORIF). The adverse effects of conventional treatment are well documented [7,8]; experts are increasingly recognizing the necessity of applying ORIF in more complex cases. The literature expounds the numerous advantages of this technique, including lessened vertical ramus height loss, optimal temporomandibular joint (TMJ) function, painless long-term results, optimal occlusion, and the restoration of physiological conditions over a relatively short period of time [7,9]. It is evident that open treatment is associated with numerous advantages. However, it is important to note that there are also potential complications associated with this treatment method. The most common and widely discussed of these is temporary facial nerve palsy [10,11,12,13,14,15], which occurs immediately postoperatively ranging from approximately 10% [13] to approximately 50% [14,15,16]. However, it is also worth mentioning other possible complications, such as wound infection [17], salivary fistula [18], sialocoele, Frey’s syndrome [19], hematoma, great auricular nerve disturbance, and unesthetic facial scarring [20,21,22,23]. It was noted that the type and incidence of postsurgical complications may be associated with the surgical approach employed [18,24]. Many ORIFs for mandibular condyles are carried out via transparotid approaches. The safety protocol requires dissection of the parotid gland parenchyma to identify the branches of the facial nerve that run level with the condylar fracture (Figure 1). This ensures preserved facial muscle function after osteosynthesis, but also constitutes a risk factor of salivary fistula formation [19].

A postoperative salivary fistula is a rare but especially unpleasant complication. The condition results in a significant reduction in patient quality of life, causing suffering. As reported in the literature, fistula incidence ranges from 4.3% [21] to 10.7% [25]. However, in the field of mandibular condylar process surgery, it is particularly rarely discussed, which is surprising given the importance of the topic. In many studies [21,25,26], only the frequency of salivary fistulas is mentioned. However, it is essential to know how to examine and treat this condition when it occurs. Salivary fistula formation in a surgical wound is a complication that may arise after a procedure. Post-surgically, the wound suture may leak, thus allowing saliva to escape. This has been observed to promote fistula formation. Saliva leakage through the wound can be prevented through the watertight suturing of the salivary gland capsule and shallow-lying tissues (Figure 1). The condition is characterized by an abnormal connection between the salivary gland and the skin surface. The cause of the leak can be established through a comprehensive physical examination of the surgical wound/scar. During diagnosis, patient observations are also important. If leakage increases during meals, it is highly probable that this is a salivary fistula. When a salivary leak is confirmed and its precise location identified, the treatment plan may include non-surgical and/or surgical interventions [26,27,28].

Following a detailed analysis of the extant literature, the authors concluded that the current knowledge does not describe the problem exhaustively and that further research is needed to provide a more comprehensive understanding. The present study was designed to investigate the incidence of postoperative salivary fistulas in patients who underwent open reduction and internal fixation (ORIF) for mandibular condylar fractures. In addition, it aimed to analyze the therapeutic outcomes of different management strategies in order to provide a more comprehensive understanding of the effectiveness of available treatment modalities.

## 2. Materials and Methods

Retrospective material was obtained from the medical records of all patients admitted to the Department of Maxillofacial Surgery at the Medical University of Lodz (Poland) who underwent surgical treatment for a mandibular condylar process fracture between 2017 and 2024. Institutional approval was granted before data collection and the research also obtained approval from the Bioethics Committee (RNN/104/25/KE). This study was reported in accordance with the Strengthening the Reporting of Observational Studies in Epidemiology (STROBE) guidelines [29]. As this study was conducted in a retrospective way, the sample size was created to include all the patients who met the inclusion criteria.

The inclusion criteria that were employed in this study were as follows:

- A diagnosis of mandibular condylar process fracture;
- ORIF treatment;
- Complete medical history and personal data;
- Patients attending follow-up appointments.

The exclusion criteria were as follows:

- Close treatment;
- Incomplete medical history;
- Lack of follow-up appointments;
- Patients with a history of cancer of the head and neck region.

The data were collected by one surgeon. The final data set comprised 395 patients aged 11–88 years, with a median age of 37 years old. Patients were observed during a six-month follow-up. Fractures were classified based on the three most widely recognized classifications (Kozakiewicz’s, Neff’s, and Loukota’s). Six diagnoses of mandible condylar fracture could be established: base, low-neck, and high-neck fractures and head fracture types A, B, and C [30,31,32].

A comprehensive data set was obtained from patients, including demographic variables such as age, sex, and body mass index (BMI). The surgical intervention data included details regarding delays (in days), surgery duration (in minutes), and use of fixation materials. Preoperative characteristics, including hemoglobin and white blood cell levels, were also documented. The analysis considered the patients’ place of residence, the type of condylar process fracture, the season of the year during surgery, whether the patient had been referred from another medical center, the surgical approach, co-morbidities, postoperative C-reactive protein level (CRP), the presence of preauricular skin desensitization and facial nerve function in seven examinations during follow-up (immediately postoperatively and after 1, 2, 3, 4, 5, and 6 months).

In our center, BMI is calculated by dividing a person’s weight in kilograms by their height in meters squared (BMI = weight (in kg)/height^2^ (in m^2^)). BMI categorizes a person as underweight (BMI < 18.5), normal weight (BMI 18.5–24.9), overweight (BMI 25.0–29.9), and obese (BMI ≥ 30). Normal white blood cell (WBC) levels are in the range of 4000–10,000/µL for adults. CRP values below 5 mg/L are reported as normal, levels above 10 mg/L are a sign of inflammatory process, and levels above 100 mg/L are treated as a serious inflammatory/immunologic response [33].

**Figure 2:**
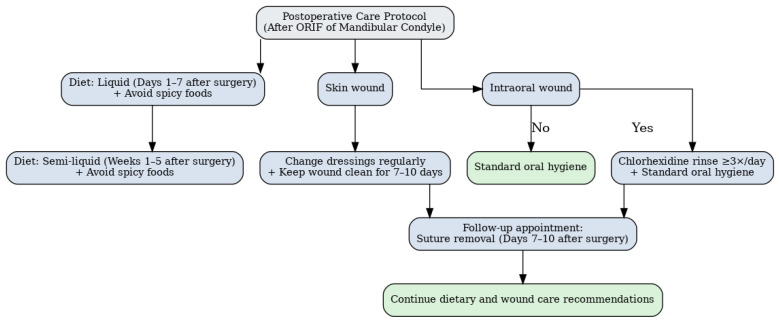
Standard postoperative care and recommendation protocol following ORIF of mandibular condylar fractures.

In all 395 cases of mandibular condylar fractures managed with ORIF, identical postoperative instructions were provided. Patients were advised to follow a liquid diet for the first 7 days, followed by a semi-liquid diet for 5 weeks, while avoiding spicy foods. The dressings on the skin wound should be changed regularly and kept clean for 7 days whenever a long-term wound wear cannot be applied. A subsequent appointment should be established for suture removal between days 7 and 10 post-procedure. In the event of intraoral wounds, it is recommended that a chlorhexidine rinse is used at least three times a day, in addition to standard oral hygiene practices (Figure 2).

**Figure 3:**
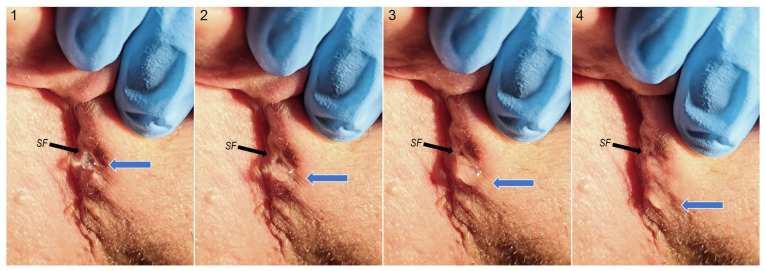
The appearance of a salivary fistula (SF) in the retromandibular region (black arrow). The next four pictures show the outflow of saliva (blue arrow) through the skin fistula formed in the surgical scar (retromabdibular approach). This is a patient being treated with silver nitrate, cholinolytic drug, and pressure dressing therapy, and following a tasteless diet. The numbers 1, 2, 3, and 4 correspond to the sequential stages of saliva outflow.

Salivary fistulas were detected by surgeons during patient examination in the follow-up period or noted by patients during self-assessment. In accordance with the severity of salivary leakage (Figure 3), complication severity was established using a three-stage scale (Table 1). Patients exhibiting salivary fistulas were administered one of three treatment modalities:

1. Disinfection, a tasteless diet, and pressure dressing;
2. Chemical cauterization with a 10% solution of AgNO_3_, a cholinolytic drug, a tasteless diet, and pressure dressing;
3. Surgical treatment—plastic surgery of the fistula.

The treatment plan was determined by the surgeon following fistula assessment. The choice of method depended on the severity of salivary leakage, which was classified using a three-level qualitative scale of saliva outflow intensity (Table 1). The characteristics of the intensity of salivary leakage are as follows:

1. Slight: For the first degree on the salivary leakage scale, manifestations are mostly only reported by patients. The amount of saliva flow is barely visible, appearing as single drops on the aperture area, especially during eating. The skin around the aperture looks healthy, with no signs of irritation or inflammation. The patient’s quality of life is not significantly impaired, although some patients report esthetic dissatisfaction.

2. Moderate: For the second degree on the salivary leakage scale, manifestations are clinically visible during examination; the surgeon detects moisture on the aperture area, and the patient reports a continuous salivary flow, especially during eating and sometimes when talking. A dressing needs to be worn on the salivary aperture skin. The skin around the aperture may be irritated, but often remains unchanged or is only slightly altered. This complication influences the patient’s quality of life because of the constant need to wear dressings, and because of the discomfort and embarrassment experienced during ordinary daily activities.

3. Profuse: For the third degree on the salivary leakage scale, manifestations are clearly visible during clinical examination and the surgeon detects a significant amount of saliva. The patient reports a constant flow of saliva during the day and night. The dressing on the aperture area needs to be changed many times per day. In many cases, the skin around the aperture is damaged and painful due to constant irritation. Signs of inflammation may also be visible. Quality of life is significantly impaired due to the tremendous salivary flow, which makes it difficult to lead a normal life and deal with everyday activities.

In cases of minor saliva leakage, the most conservative approach was adopted. This entailed the patient undergoing fistula disinfection, following a tasteless diet, and applying a pressure dressing. In the event of moderate saliva flow and a medium-sized fistula aperture, a combination of measures was employed, including a tasteless diet, pressure dressing, chemical cauterization, and the administration of cholinolytic drugs. Cases where there was a high amount of saliva flow and a large fistula outlet qualified for a two-layer salivary fistula surgical procedure.

Due to there being several different surgeons, the salivary fistula was disinfected using four types of rinsing solution; the choice of rinsing solution depended on the attending doctor’s preferences. These were a chlorhexidine solution, an octenidine solution, disinfectants comprising nanosilver and solutions including sodium hypochlorite (NaClO_3_) and hypochlorous acid (HClO). The fistula skin area was chemically cauterized using a 10% argentum nitricum—otherwise known as silver nitrate—solution.

Surgical plasty of the fistula involves the removal of the pathological channel in soft tissues and the restoration of tissue continuity. Prior to undergoing surgery, comprehensive diagnostic tests are conducted to ascertain the characteristics of the fistula and its underlying etiology. Furthermore, it is essential to provide the patient with comprehensive information regarding the surgery itself, the potential risks of complications, and the expected recovery period. In the event of a surgical wound in the parotid gland area being revised, precise tissue preparation is conducted to avoid facial nerve damage. The dissected tissues should then be sutured securely and in layers. The initial layer of the suture is the accurately sutured periauricular fascia; the second layer is the subskin suture. It is crucial to ensure that the suture lines of these two tissue layers do not run on top of each other. The suture lines should be laterally offset in order to reduce the likelihood of subsequent postoperative wound healing failure and salivary fistula return.

Statistical analyses were conducted using Statgraphics Centurion 18 (Statgraphics Technologies Inc., The Plains, VA, USA). The procedure included tests of normality and a paired-samples sign test to assess time-dependent variations. The effect of the qualitative variable on salivary fistula formation was examined using the Kruskal–Wallis test, and regression analysis was applied to explore associations between quantitative variables. Moreover, logistic regression was performed to ascertain independent risk factors for salivary fistula complication after osteosynthesis. For this. Then, due to the identification of a small number of independent risk factors, the authors sought composite factors via factor analysis. Reducing the number of variables describing the study population makes it easier to interpret the data in a multidimensional environment. A *p*-value of less than 0.05 was interpreted as statistically significant.

## 3. Results

The statistical analysis revealed that 5.8% of patients treated with ORIF developed postoperative complications in the form of a salivary fistula (Table 2). Within the group of 23 patients, the male-to-female ratio was 22:1. Those who presented with salivary fistulae were aged between 19 and 62 years. Patients with this complication manifested four types of mandibular condylar fracture: condylar head fracture types B and C, and low-neck and base fractures (no case with high-neck fracture developed a fistula). In the course of the primary surgical procedures for condyle fracture, various surgical approaches were employed, including the following: retromandibular, extended retromandibular, preauricular, and extended preauricular. Treatment involved one of four distinct fixation types: one straight plate, two straight plates, an XCP plate, an ACP plate, or compressive screws (manufactured by ChM, Juchnowiec Kościelny, Poland, www.chm.eu access date 24 July 2025). The time interval from surgery completion (ORIF) to the manifestation of the salivary fistula was found to be 6–32 days (median: 13 days). The duration of fistula treatment varied between 2 and 41 days, with a median value of 10 days. All treated patients achieved fistula recovery. Three different types of treatment protocols were implemented (the method depended on the intensity of saliva efflux): (1) disinfection, a tasteless diet, and pressure dressing; (2) chemical cauterization by 10% solution of AgNO_3_, a cholinolytic drug, a tasteless diet, and pressure dressing; and (3) surgical treatment (plastic surgery). The number of procedures required to close the fistula ranged from one to three. Of the 23 patients, 19 presented salivary fistula; while some were successfully treated with one closure attempt, 3 required two attempts, and 1 required three attempts to achieve successful outcome in managing the salivary fistula.

**Figure 4:**
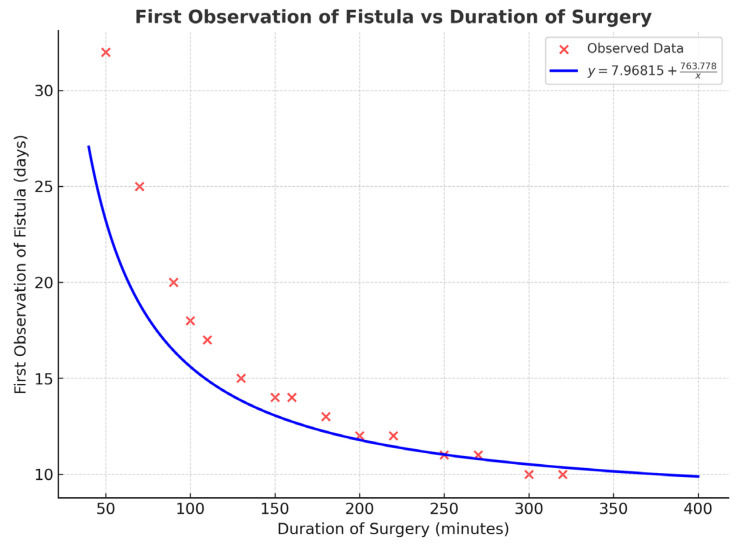
The relation between the time of the fistula diagnosis (days) and the duration of surgical procedure of the mandibular condyle fixation (minutes); *p* = 0.045.

This investigation has shown that some factors influence how late after surgery a salivary fistula might appear. The longer the surgical procedure takes, the earlier the fistula will appear (correlation coefficient, CC = 0.478, *p* = 0.045) during the postoperative follow-up period (Figure 4).

The research indicated three factors (white blood cell levels, CRP and BMI) that influence fistula treatment duration. Moderately elevated white blood cell levels (*p* = 0.0006) promote faster salivary fistula withdrawal (Table 3). CRP levels that are moderately elevated after surgery are related faster salivary fistula healing (*p* = 0.0003) (Table 3). For a salivary fistula, the length of the treatment period is also related to BMI (*p* = 0.0467), where the lower BMI, the longer treatment (Table 3).

**Figure 5:**
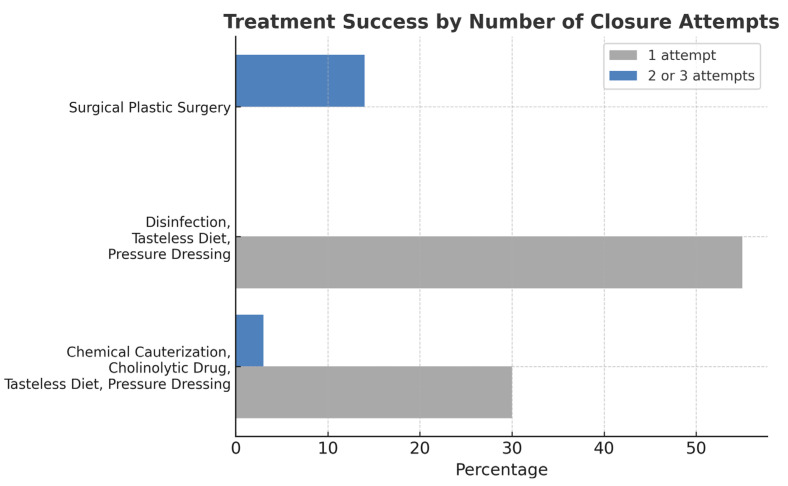
Demonstration of the effectiveness of salivary fistula treatment. A comparison of the effectiveness of treatment in terms of the number of treatment attempts needed to completely cure the salivary fistula using one of the utilized methods. The vertical axis shows the treatment method, while the horizontal axis shows the number of treated fistulas [in percentage]; *p* = 0.0002.

The most efficacious method of closing salivary fistulas is disinfection, the application of pressure dressing and a tasteless diet. In half of the patients observed, this was sufficient to permanently close the fistula (*p* = 0.0002) (Figure 5). This treatment has been shown to result in fistula closure within a period of 10 d (Table 4). The increased number of treatment attempts prolongs salivary fistula treatment duration (*p* < 0.05).

Moreover, a comprehensive statistical analysis was conducted on a range of factors potentially impacting postoperative salivary fistula formation. These factors encompassed demographic details such as age and BMI; details of the surgical intervention including any delay, surgery duration, surgical approach, and intraoperative salivary gland management (transparotid or bypassing approach) (Table 5); and preoperative characteristics such as hemoglobin and white blood cell levels. The analysis also considered the patient’s place of residence, reason for the injury, the type of condylar process fracture, the season of the year during surgery, patient referral from another center, patient intoxicant use, the presence of preauricular skin desensitization and facial nerve function in seven examinations during the follow-up. However, upon statistical evaluation, these factors were found to be statistically insignificant in terms of their impact on the occurrence of postoperative salivary fistulas; *p* > 0.05.

We next focused on identifying independent risk factors for salivary fistula formation after surgical treatment for mandibular condylar process fracture. A comprehensive investigation involving 27 potential factors was conducted utilizing logistic regression. Male gender (*p* = 0.009) was identified as the only one independent risk factor for salivary fistula formation. This study found that males have a seven times higher risk of developing a salivary fistula after ORIF for a condylar fracture than females (Table 6).

The variables investigated as potential risk factors were as follows: age, BMI, any surgery delay, surgery duration, patient co-morbidities, preoperative hemoglobin level, preoperative white blood cell count, number of screws used for surgical stabilization, immediate postoperative facial nerve function, facial nerve function during follow-up at 1, 2, 3, 4, 5, and 6 months after surgery, sex, patient location, reason for injury, use of intoxicants, diagnosis of injury, type of condylar fracture, season of the year during injury, referral from other centers, surgical approach, screw material, type of fixation material, and occurrence of preauricular skin desensitization. Logistic regression only indicated sex as an independent risk factor.

**Figure 6:**
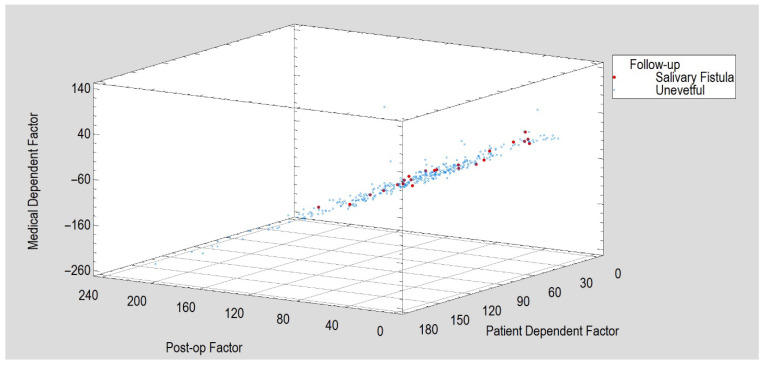
Evaluation of salivary fistula incidents depending on three calculated factors aggregating information from 8 variables (age, BMI, co-morbidity, any surgery delay, surgery duration and House–Brackmann Scale 00M, House–Brackmann Scale 01M, House–Brackmann Scale 02M).

**Figure 7:**
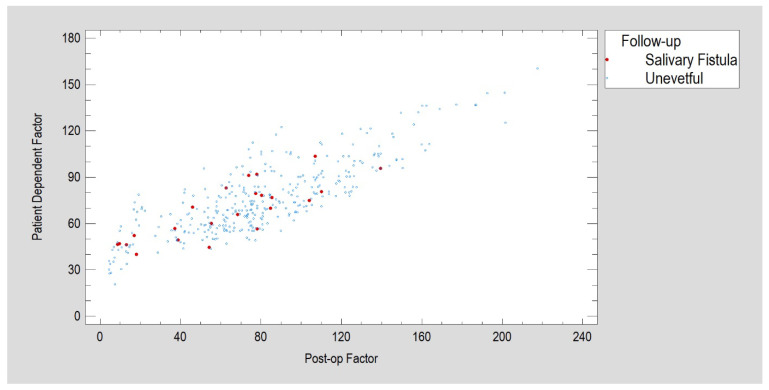
Post-surgical salivary fistulas in patients less affected by early facial nerve dysfunction (post-op factor < 150) in a subgroup of younger patients and those with lower internal patient involvement (patient-dependent factor < 110).

The variables selected for factor analysis were age, BMI, co-morbidity, any surgery delay, surgery duration and magnitude of facial nerve dysfunction, immediately after osteosynthesis and 1 and 2 months post-op (House–Brackmann Scale 00M, House–Brackmann Scale 01M, House–Brackmann Scale 02M). This selection was dictated by sampling adequacy, as expressed by the Kaiser–Meyer–Olkin (KMO) measure. A test score of 0.7 was obtained for this input data set. For factorization to be worthwhile, KMO should be at least 0.6. Thus, factorization was likely to provide interesting information about any underlying factors. Next, three factors were extracted that had an Eigenvalue greater than 1. Together, the factors expressed 71.4% of the variability contained in the eight input variables. We determined three equations for factor calculation after matrix rotation (Figure 6 and Figure 7). Rotation was performed in order to simplify factor explanations. The first rotated factor has the following equation (the variable values were standardized by subtracting their means and dividing by their standard deviations):

Post-op Factor = 0.178046 × Age − 0.142933 × BMI + 0.123149 × Delay of Surgery + 0.415526 × Duration of Surgery + 0.0495633 × Co-Morbidity + 0.965919 × House–Brackmann Scale 00M + 0.978027 × House–Brackmann Scale 01M + 0.957083 × House–Brackmann Scale 02M(1)

This factor highlights the importance of characteristics that describe the patient’s postoperative state.

The second rotated factor has the following equation:

Patient Dependent Factor = 0.825884 × Age + 0.454182 × BMI + 0.0747151 × Delay of Surgery + 0.190828 × Duration of Surgery + 0.766203 × Co-Morbidity − 0.00920252 × House–Brackmann Scale 00M + 0.0260572 × House–Brackmann Scale 01M + 0.0801639 × House–Brackmann Scale 02M(2)

This factor mainly describes a patient’s pre-surgical condition.

The third rotated factor has the following equation:

Medical Dependent Factor = − 0.120654 × Age + 0.27525 × BMI + 0.871126 × Delay of Surgery − 0.532189 × Duration of Surgery − 0.052594 × Co-Morbidity − 0.00227824 × House–Brackmann Scale 00M − 0.0479474 × House–Brackmann Scale 01M − 0.0618094 × House–Brackmann Scale 02M(3)

This factor most strongly depends on—and thus indicates the importance of—variables dependent on the activities of the maxillofacial surgery department.

During follow-up, a Kruskal–Wallis test for post-op and medical dependent factors (incidents of fistula creation) revealed that, in the group of patients with fistulas, the former reached a median of 68 versus in 77 in the healthy group (a statistically significant difference, *p* < 0.05); in terms of the medical dependent factor, the median was 60 in the fistula group versus 79 in the healthy group (*p* < 0.05). The patient-dependent factor value did not differ between the uneventful recovery and fistula formation groups (medians of 74 and 70, respectively).

## 4. Discussion

Salivary fistula formation generally results from disruption of the salivary duct or injury to glandular tissue, with subsequent leakage occurring through the surgical suture line. Depending on its location, a salivary fistula can be classified as either cutaneous or intraoral. Only cutaneous fistulas require treatment due to the long-term inconvenience for the patient and due to esthetic reasons. Constant skin irritation from the flowing saliva can cause pathological skin lesions. Salivary fistulas mainly affect the parotid glands [18,34,35].

Analyzing the results, male sex was revealed to be an independent risk factor for the formation of salivary fistulas as a complication of surgical treatment for condylar process fractures. A retrospective cohort study conducted by Al-Taki et al. examined the effect of gender on postoperative morbidity and mortality. The results indicated that the female gender was less associated with complications and mortality after surgery than the male gender [36]. This is consistent with our observations. Our study is the first report in the available scientific literature to demonstrate a statistical relationship between sex and salivary fistula. Admittedly, there are only a few papers in this area; previous studies have shown no relationship between the effect of patient sex on salivary fistula formation [37,38,39]. Our results may be explained by the generally poorer compliance [40,41] and postoperative self-care of male patients. Our hypothesis is based on observations of our patients. However, results could be different for patients from different demographics. This phenomenon was discussed in Baker’s paper, which presented the thesis that males tend to take more risks with their health and are less likely than females to be aware of disease symptoms [42]. The postoperative recommendations highlight the importance of a soft diet; however, based on studies that revealed that males tend to have worse dietary habits, the authors can conclude that an improper diet after surgery could also be linked to the higher incidence of salivary fistula formation in male patients. The findings of Imamura et al. [43] are in line with this hypothesis, as their research indicated that females tended to have better dietary habits than males. In many studies, males are noted to maintain poorer hygiene [44,45,46], which could also be connected to complications in postoperative wound healing. Males also tend to not seek medical advice when the first symptoms of disease or treatment failure appear. This is evident in demographic statistics showing that males live shorter lives than females in all European countries [47].

It is worth nothing that extended surgical procedure duration can be associated with increased surgical intervention complexity [48]. The most complicated and hard-to-treat fractures require a proper, and often extensive, surgical approach, which is associated with greater tissue morbidity at the surgery site. The severity of tissue damage can be observed via postoperative complications during the follow-up period. In the event of long ORIF for a mandibular condylar process fracture, damaged soft tissues have been observed to result in salivary fistulas at a faster rate than in patients who have presented with the issue following short, less harmful surgical procedures. There are only a few studies in the maxillofacial surgery literature examining the influence of surgery duration on postoperative complications, particularly salivary fistulas [15,48,49,50]. However, researchers in other surgical specialties have clearly demonstrated the relationships between surgery duration and postsurgical complications [51,52].

In terms of surgical approach, our results oppose some studies. This investigation revealed that the surgical approach and the salivary gland management technique used during surgery (transparotid or bypassing the gland) were statistically irrelevant. The findings of El Sheikh et al. are consistent with our observations, as their study also did not reveal statistically significant differences between patients treated using different surgical approaches—in their study, intraoral versus retromandibular approach [53]. Nam et al. also did not detect any influence of the surgical approach on postoperative salivary fistula formation; however, they emphasized the importance of accurate and complete closure of the parotid capsule to prevent this complication [54]. The literature review demonstrates significant differences in opinion on this issue. Rozenboom et al. [21] indicated that the majority of salivary fistula complications occurred in patients treated with the transparotid approach. The complication was also most commonly associated with a retromandibular approach. The results presented by Ellis et al. [11] also indicate that retromandibular approach and the means of entry to the salivary gland are risk factors for postoperative salivary fistula formation. On the other hand, the incidence of sialocele (another parotid disorder) in the study conducted by Kulkarni et al. was most frequently associated with the periauricular approach and the second most frequent approach was the retromandibular [55]. Due to the significant differences in scientific reports in this field, the authors suggest that these discrepancies may be due to the variations in surgical techniques and tissue suturing methods used. Our hypothesis is consistent with the findings of Sikora et al., [18] who examined the transparotid approach for treating condylar fractures and noted that, the transparotid approach can increase the risk of salivary duct injury. However, their results revealed that proper surgical technique, involving careful suturing of the salivary gland capsule, helped to reduce the risk of salivary fistula formation.

Slightly elevated WBC levels are a symptom of increased immune system activity [56,57], as demonstrated by Foy et al. [58]. They can promote the body’s healing abilities [59] and speed up treatment [57,58]. This concept was hypothesized in this study, where moderately elevated WBC levels promoted salivary fistula treatment. CRP levels are also a reliable indicator of inflammation and immune system activation in the body [60]. CRP synthesis is induced by cytokines released in response to bacteria, viruses or nociceptive agents. CRP elevation can also occur during healing, and is a symptom of excessive immunological function [61]. The factors contributing to both CRP and WBC elevation are comparable [62]. It is important to remember that patient nutritional status can also be connected to immunity, healing duration and, eventually, treatment success. This condition can be examined using BMI. A lower BMI may indicate poorer nutritional status, as such patients have been shown to have lower concentrations of albumin, prealbumin and protein [63]. This results in a suppressed immune system, which is unable to respond accurately to stress. In many studies, it has been reported that patients with a low BMI are at a higher risk of experiencing postoperative complications and requiring hospital readmission. Based on our observations, the duration of fistula treatment was longer in patients with lower BMI. Similarly, the study by Paksoy and Sazak Kundi found that poor nutritional status could be a risk factor for sialoceles and salivary fistulas. This was explained by impaired healing processes and the possibility of salivary duct compromise in malnourished patients [38]. Similar findings have been noted in various surgical fields [64,65,66].

In the issue of salivary fistulae treatment duration, the study conducted by Ebenezer V. et al. [67] demonstrated similar outcomes, with the treatment period lasting a mean of three weeks. However, it should be noted that the treatment period was found to be longer when patients were treated using a more invasive method than with a mainly conservative approach. The latter is usually successful [68], often saving patients from repeated appointments and even surgery [34,69]. It is acknowledged that postoperative fistula treatment may exhibit distinct characteristics due to the varied operational methods employed in comparison to those usually employed in fistula treatment. Fistula management has a better prognosis in traumatological than in oncology patients. In the study conducted by Britt et al., the authors reported that salivary fistula treatment following parotidectomy can take up to six months [39]. Oncology patients are often compromised in terms of nutrition and immunity.

Weissler et al. demonstrated treatment methods for parotid duct injury similar those presented in our management proposition. Methods were applied in accordance with the condition severity and previous treatment effectiveness: saliva aspirations and antibiotic therapy, antisialagogue medication therapy, botulin toxin injections (in the event conservative treatment failure), and surgical intervention (for the most severe cases) [35]. One of these methods was clinically utilized by Liam and Choi, who presented a case study where a salivary fistula was successfully treated with botulin toxin type A injections [70]. In their pilot study, Cohen et al. reported that octreotide subcutaneous injections induced alteration in saliva. They suggested that this method has a potential beneficial influence on the salivary fistula healing process; however, further research is needed to confirm the clinical effectiveness of this method [71]. Many studies also have noted the use of negative pressure wound therapy (NPWT) and highlighted its beneficial effects, even in serious cases [68,72,73].

Rinsing the fistula with an appropriate disinfectant is fundamental to its management [69], as residual debris promotes bacterial growth and increases the risk of infection [74,75]. If healing is delayed, infections of the salivary glands or adjacent tissues may develop. To prevent such complications, a range of antibacterial agents can be employed in the treatment of salivary fistulas. Chlorhexidine, an effective disinfectant, is distinguished by its high efficacy and low toxicity. For the purpose of mucous membrane disinfection, solutions with a concentration of 0.1–0.5% are recommended; for skin disinfection, solutions with a concentration of 1–4% are advised. Next, Octenidine solutions demonstrate broad-spectrum antimicrobial [76,77] and high antibiofilm activity [78,79,80]. The utilization of disinfectants comprising nanosilver is gaining popularity, owing to their demonstrated effective antimicrobial properties [81,82], and thus in accelerating wound healing [83,84]. Nowadays, there are many sodium hypochlorite (NaClO_3_) and hypochlorous acid (HClO) solutions available that are formulated for use in surgical procedures. However, it is worth mentioning that the use of chloride solutions must be informed and considered. Although higher concentrations of hypochlorite solution are more effective against micropathogens, it should be noted that they may be toxic to patient tissue. The longer cells are exposed to the solution and the higher the concentration, the higher the toxicity for human cells and effectiveness against micropathogens [85,86].

Cholinolytics can be used during salivary fistula treatment to suppress glandular function during healing [77]. The action of acetylcholine is blocked, which consequently results in a reduction in salivary secretion [87]. Prior to the administration of cholinolytics for fistula treatment, it is essential to undertake a comprehensive patient medical history to ensure that the prescribed treatment does not pose a threat to their health. This group of drugs has been associated with a long list of possible side effects, including, but not limited to, tachycardia, pupil dilation and difficulty in breathing [88]. In the context of patients having undergone ORIF for condylar fracture, it is also imperative to monitor facial nerve function when treating a salivary fistula. In cases where a salivary fistula is accompanied by postoperative facial nerve dysfunction, the administration of cholinolytics is contraindicated due to their harmful effect on nerve regeneration.

In the case of chemical treatment for salivary fistulas, argentum nitricum solution can be employed for chemical cauterization. Cauterization involves a chemical acting on the granulating wound or destroying the pathological tissue [89,90]. However, while argentum nitricum is known to possess bactericidal and astringent properties [91], prolonged use of this substance has been demonstrated to result in skin or mucosa discoloration. In the field of chemical treatment, Hah et al. demonstrated a case study about congenital sialo-cutaneous fistula treatment using trichloroacetic acid and botulin toxin injection [92] for decreasing gland secretion activity.

Surgical intervention is effective in managing salivary fistulas with high saliva secretion or insufficient response to conservative therapy [18,35,69]. Blythe et al. presented a novel technique for managing hard-to-treat salivary fistulas following parotidectomy. They successfully closed a fistula using an acellular dermal matrix as a resorbable barrier membrane. This technique may therefore be considered by surgeons and researchers as a new method for managing clinically complicated fistulas [93].

It is acknowledged that this study is not without its limitations. The data were collected by one surgeon from a single medical center, what can be connected with limited diversity of patient demographics and local treatment protocols. Therefore, the possibility of generalizing the results to other institutions may be limited. The retrospective character of this paper resulted in a small sample size, risk of missing data or errors in medical records, and differences in the medical histories reported by different surgeons over the seven-year period. The surgical procedures were conducted by surgeons with different levels of experience. Various suture materials and surgical techniques were used, as well as different rinsing solutions. Due to the limited studies describing this issue and the impossibility of conducting a substantive discussion of the field, our investigation is pioneering, and the results should be treated more as hypotheses rather than theses.

We suggest that further research should concentrate on repeating these investigative methods on larger cohorts from different societies and varying patient demographics. This would help to strengthen the validity and reliability of clinical results in this surgical field. Research and development into more innovative methods of treating salivary fistulas, especially in more complicated cases, is also necessary.

## 5. Conclusions

Salivary fistula after ORIF for condylar process fractures is a rare complication, occurring more frequently in males. This condition can typically be managed within a short treatment period (median: 10 days), most often with conservative, minimally invasive methods such as disinfection, pressure dressing, and a tasteless diet. Moderately elevated WBC and CRP levels appear to facilitate healing, whereas lower BMI may delay recovery. Further studies are required to confirm these findings due to the limitations of the present study.

## Figures and Tables

**Table 1:** The scale of the salivary fistula severity in accordance with the intensity of salivary leakage and the dedicated scheme of treatment used by our center. The algorithm was implemented at the beginning of treatment. In cases where effectiveness was lacking, the algorithm was modified.

Intensity of Salivary Leakage	Clinical Manifestation and/or Patient’s Observations	Treatment Method
Slight	Clinically visible aperture in the skin of the affected area, clinically no leakage of saliva; patient reports history of saliva flow	Disinfection, tasteless diet, pressure dressing
Moderate	Clinically visible moist aperture in the skin of the affected area	Chemical cauterization, cholinolytic drug, tasteless diet, pressure dressing
Profuse	Clinically visible aperture in the skin of the affected area, clinically visible salivary flow	Plastic surgery procedure

Slight: the occasional appearance of saliva drops; moderate: chronic saliva flow in small amounts, especially during meals; profuse: constant saliva flow in large amounts during the day and night.

**Table 2:** Preoperative variables analyzed as potential risk factors for salivary fistula formation during the follow-up period. Results of the statistical analysis are summarized in the table.

Variable	Complication	Mean ± SD	*p*-Value
Age [years]	No Salivary Fistula	40.16 ± 16.54	0.548
	Salivary Fistula	40.43 ± 11.90	
BMI [kg/m^2^]	No Salivary Fistula	23.04 ± 4.29	0.922
	Salivary Fistula	22.89 ± 3.55	
Total Mandibular Fractures	No Salivary Fistula	2.01 ± 0.82	0.389
	Salivary Fistula	1.87 ± 0.81	
Delay of Surgery Delay [days]	No Salivary Fistula	8.76 ± 11.89	0.990
	Salivary Fistula	8.39 ± 6.86	
Co-Morbidity	No Salivary Fistula	0.50 ± 0.87	0.254
	Salivary Fistula	0.30 ± 0.63	
Hemoglobin Level [g/dL]	No Salivary Fistula	**14.02 ± 1.71**	**0.02**
	Salivary Fistula	**14.83 ± 1.21**	
White Blood Cell Count [10^9^/L]	No Salivary Fistula	**7.94 ± 3.00**	**0.01**
	Salivary Fistula	**9.80 ± 3.99**	

Statistically, significant differences (*p* < 0.05) between the variants of the variable are indicated in bold.

**Table 3:** The relation between salivary fistula treatment duration and patient’s BMI level and postoperative levels of WBC and CRP. All variables reached statistical significance (*p* < 0.05).

Variable	Correlation Coefficient	*p*-Value
WBC [10^9^/L]	0.66	***p* = 0.0006**
CRP [mg/L]	0.76	***p* = 0.0003**
BMI [kg/m^2^]	0.43	***p* = 0.0467**

**Table 4:** Treatment durations (in days) using different methods, including the most effective one: disinfection, tasteless diet, and pressure dressing.

Treatment Method	No of Patients	Mean ± SD	Median and IQR
Disinfection, tasteless diet, pressure dressing	12	11.75 ± 9.41	10 (7–41)
Chemical cauterization, cholinolytic drug, tasteless diet, pressure dressing	8	13.12 ± 11.56	7.5 (2–35)
Surgical plastic surgery	3	13.33 ± 7.09	12 (7–21)
**Total**	**23**	**12.43 ± 9.59**	**10 (2–41)**

SD—standard deviation; IQR—interquartile range.

**Table 5:** Evaluation of surgical approaches in relation to parotid gland management (bypassing vs. transparotid). Statistical analysis revealed no significant differences between the two methods.

Variable	Bypassing Approach (n = 39)	Transparotid Approach (n = 356)	*p*-Value
Type of fixation			*p* = 0.488
Dedicated plate	13 (33%)	137 (38.5%)	
Standard plate	26 (67%)	219 (61.5%)	
Diagnosis			*p* = 0.202
No salivary fistula	39 (100%)	333 (93.5%)	
Salivary fistula	0	23 (6.5%)	

**Table 6:** Results for the only independent risk factor identified for salivary fistula formation. The vertical axis represents patient sex. The model indicates that female sex is associated with recovery, whereas male sex is linked to an increased risk of salivary fistula development.

Estimated Regression Model (Maximum Likelihood)			
Parameter	Estimate	Standard Error	Estimated Odds Ratio
Constant	−2.544	0.221	
Sex = Female ^1^	−1.978	1.030	0.138
Analysis of Deviance			
Source	Deviance	Df	*p*
Model	6.766	1	0.0093 ^2^
Residual	168.66	393	1
Total (corr.)	175.43	394	

^1^ Sex, predicting uneventful post-operational healing. ^2^ Because the *p*-value for the model in the Analysis of Deviance table is less than 0.05, there is a statistically significant relationship between the variables at the 95.0% confidence level. In addition, the *p*-value for the residuals is greater than or equal to 0.05, indicating that the model is not significantly worse than the best possible model for these data at the 95.0% or higher confidence level.

## Data Availability

The data presented in this study are available on request from the corresponding author.

## Abbreviations

The following abbreviations are used in this manuscript:

ORIF	Open Rigid Internal Fixation
BMI	Body Mass Index
CRP	C-reactive Protein Level
WBC	White Blood Cells

## References

[1] Wusiman P., Maimaitituerxun B., Guli Saimaiti A., Moming A. (2020). Epidemiology and Pattern of Oral and Maxillofacial Trauma. J. Craniofac. Surg..

[2] Ascani G., Di Cosimo F., Costa M., Mancini P., Caporale C. (2014). Maxillofacial Fractures in the Province of Pescara, Italy: A Retrospective Study. ISRN Otolaryngol..

[3] Kozakiewicz M., Walczyk A. (2023). Current Frequency of Mandibular Condylar Process Fractures. J. Clin. Med..

[4] Wayiso G.T., Tola F.S., Erba M.S., Desta D. (2025). Etiology and Pattern of Maxillofacial Fractures among Patients Who Visited Jimma Medical Center Dental Clinic, Jimma, Southwest Ethiopia. Medicine.

[5] Kozakiewicz M., Zieliński R., Krasowski M., Okulski J. (2019). Forces Causing One-Millimeter Displacement of Bone Fragments of Condylar Base Fractures of the Mandible after Fixation by All Available Plate Designs. Materials.

[6] Sikora M., Chęciński M., Nowak Z., Chęcińska K., Olszowski T., Chlubek D. (2021). The Use of Titanium 3D Mini-Plates in the Surgical Treatment of Fractures of the Mandibular Condyle: A Systematic Review and Meta-Analysis of Clinical Trials. J. Clin. Med..

[7] Kolk A., Scheunemann L.M., Grill F., Stimmer H., Wolff K.D., Neff A. (2020). Prognostic Factors for Long-Term Results after Condylar Head Fractures: A Comparative Study of Non-Surgical Treatment versus Open Reduction and Osteosynthesis. J. Cranio-Maxillofac. Surg..

[8] Valiati R., Ibrahim D., Abreu M.E., Heitz C., de Oliveira R.B., Pagnoncelli R.M., Silva D.N. (2008). The Treatment of Condylar Fractures: To Open or Not to Open? A Critical Review of This Controversy. Int. J. Med. Sci..

[9] Neff A. (2019). Open Reduction and Internal Fixation in Temporomandibular Joint Traumatology: Current Concepts and Future Perspectives. Stomatol. Dis. Sci..

[10] Bhutia O., Kumar L., Jose A., Roychoudhury A., Trikha A. (2014). Evaluation of Facial Nerve Following Open Reduction and Internal Fixation of Subcondylar Fracture through Retromandibular Transparotid Approach. Br. J. Oral Maxillofac. Surg..

[11] Ellis E., McFadden D., Simon P., Throckmorton G. (2000). Surgical Complications with Open Treatment of Mandibular Condylar Process Fractures. J. Oral Maxillofac. Surg..

[12] Ghezta N.K., Ram R., Bhardwaj Y., Sreevidya S., Sharma M., Bhatt R. (2021). Operator Experience and Fracture Location Affects the Rate of Facial Nerve Injury in Condylar Fractures: An Analysis of 89 Cases. J. Oral Maxillofac. Surg..

[13] Neff A., Neff F., Kolk A., Horch H.H. (2001). Risiken und perioperative Komplikationen bei offenen gelenkchirurgischen Eingriffen. Dtsch. Zahnärztl. Z..

[14] Tandon S., Verma V., Rashid M., Srivastava S., Singh A.K., Sharma N.K. (2022). Is the Facial Nerve at Risk Following Surgical Correction of Mandibular Condylar Fracture: A Systematic Review and Meta-Analysis. Natl. J. Maxillofac. Surg..

[15] Agier P., Kozakiewicz M., Tyszkiewicz S., Gabryelczak I. (2025). Risk of Permanent Dysfunction of Facial Nerves After Open Rigid Internal Fixation in the Treatment of Mandibular Condylar Process Fracture. Med. Sci..

[16] Anehosur V., Kulkarni K., Shetty S., Kumar N. (2019). Clinical outcomes of endoscopic vs retromandibular approach for the treatment of condylar fractures-a randomized clinical trial. Oral Surg. Oral Med. Oral Pathol. Oral Radiol..

[17] Balasundram S., Kovilpillai F.J., Royan S.J., Ma B.C., Gunarajah D.R., Adnan T.H. (2020). A 4-Year Multicentre Audit of Complications Following ORIF Treatment of Mandibular Fractures. J. Maxillofac. Oral Surg..

[18] Sikora M., Olszowski T., Sielski M., Stąpor A., Janiszewska-Olszowska J., Chlubek D. (2015). The use of the transparotid approach for surgical treatment of condylar fractures—Own experience. J. Craniomaxillofac. Surg..

[19] Sverzut C.E., Trivellato A.E., Sverzut A.T., de Moraes M. (2004). Frey’s Syndrome after Condylar Fracture: Case Report. Braz. Dent. J..

[20] Bouchard C., Perreault M.H. (2014). Postoperative Complications Associated with the Retromandibular Approach: A Retrospective Analysis of 118 Subcondylar Fractures. J. Oral Maxillofac. Surg..

[21] Rozeboom A.V.J., Dubois L., Boss R.R.M., Spijker R., de Lange J. (2018). Open Treatment of Condylar Fractures via Extraoral Approaches: A Review of Complications. J. Cranio-Maxillofac. Surg..

[22] Al Hasani K.M., Bakathir A.A., Al-Hashmi A.K., Albakri A.M. (2024). Complications of Open Reduction and Internal Fixation of Mandibular Condyle Fractures in Oman. Sultan. Qaboos. Univ. Med. J..

[23] García-Guerrero I., Ramírez J.M., Gómez de Diego R., Martínez-González J.M., Poblador M.S., Lancho J.L. (2018). Complications in the Treatment of Mandibular Condylar Fractures: Surgical versus Conservative Treatment. Ann. Anat..

[24] Lu X.W., Liu X.G. (2022). Risk of Complications with Retromandibular Transparotid vs. Anteroparotid Approach for Condylar Fractures: A Systematic Review and Meta-Analysis. Eur. Rev. Med. Pharmacol. Sci..

[25] Kim B.K., Kwon Y.D., Ohe J.Y., Choi Y.H., Choi B.J. (2012). Usefulness of the Retromandibular Transparotid Approach for Condylar Neck and Condylar Base Fractures. J. Craniofac. Surg..

[26] Vesnaver A., Ahčan U., Rozman J. (2012). Evaluation of Surgical Treatment in Mandibular Condyle Fractures. J. Cranio-Maxillofac. Surg..

[27] Belcastro A., Reed W., Puscas L. (2022). The Management of Salivary Fistulas. Semin. Plast. Surg..

[28] Gurukeerthi B., Thiagarajan S., Chidambaranathan N., Chaukar D. (2022). Parotid fistula and/or salivary collection: An underrecognized and underreported preventable complication following surgery for oral cancer. Oral Surg. Oral Med. Oral Pathol. Oral Radiol..

[29] von Elm E., Altman D.G., Egger M., Pocock S.J., Gotzsche P.C., Vandenbroucke J.P., STROBE Initiative (2007). The Strengthening the Reporting of Observational Studies in Epidemiology (STROBE) Statement: Guidelines for Reporting Observational Studies. Lancet.

[30] Kozakiewicz M. (2019). Classification Proposal for Fractures of the Processus Condylaris Mandibulae. Clin. Oral Investig..

[31] Neff A., Cornelius C.P., Rasse M., Torre D.D., Audigé L. (2014). The Comprehensive AOCMF Classification System: Condylar Process Fractures—Level 3 Tutorial. Craniomaxillofac. Trauma Reconstr..

[32] Loukota R.A., Rasse M. (2010). Nomenclature/Classification of Fractures of the Mandibular Condylar Head. Br. J. Oral Maxillofac. Surg..

[33] Cederholm T., Jensen G.L., Ballesteros-Pomar M.D., Blaauwe R., Correia M.I.T.D., Cuerda C., David C., Fukushima R., Gautier J.B.O., Gonzalez M.C. (2024). Guidance for Assessment of the Inflammation Etiologic Criterion for the GLIM Diagnosis of Malnutrition: A Modified Delphi Approach. Clin. Nutr..

[34] Hyman J., Disa J.J., Cordiero P.G., Mehrara B.J. (2006). Management of Salivary Fistulas after Microvascular Head and Neck Reconstruction. Ann. Plast. Surg..

[35] Weissler J.M., Mohamed O., Gryskiewicz J.M., Chopra K. (2022). An Algorithmic Approach to Managing Parotid Duct Injury Following Buccal Fat Pad Removal. Aesthetic Surg. J. Open Forum.

[36] Al-Taki M., Sukkarieh H.G., Hoballah J.J., Jamali S.F., Habbal M., Masrouha K.Z., Abi-Melhem R., Tamim H. (2018). Effect of Gender on Postoperative Morbidity and Mortality Outcomes: A Retrospective Cohort Study. Am. Surg..

[37] Liu Y., Liu M., Wang Y., Li W., Guo C., Zhang Y. (2022). Predictors of Sialocele or Salivary Fistula after Partial Superficial Parotidectomy for Benign Parotid Tumor: A Retrospective Study. J. Oral Maxillofac. Surg..

[38] Paksoy Z.B., Sazak Kundi F.C. (2024). Impact of the Prognostic Nutritional Index on the Development of Sialocele or Salivary Fistula After Parotidectomy. Clin. Otolaryngol..

[39] Britt C.J., Stein A.P., Gessert T., Pflum Z., Saha S., Hartig G.K. (2017). Factors Influencing Sialocele or Salivary Fistula Formation Postparotidectomy. Head Neck.

[40] Oehl M., Hummer M., Fleischhacker W.W. (2000). Compliance with Antipsychotic Treatment. Acta Psychiatr. Scand. Suppl..

[41] Nosé M., Barbui C., Tansella M. (2003). How Often Do Patients with Psychosis Fail to Adhere to Treatment Programmes? A Systematic Review. Psychol. Med..

[42] Backer P. (2016). Men’s Health: An Overlooked Inequality. Br. J. Nurs..

[43] Imamura F., Micha R., Khatibzadeh S., Fahimi S., Shi P., Powles J., Mozaffarian D. (2015). Dietary Quality among Men and Women in 187 Countries in 1990 and 2010: A Systematic Assessment. Lancet Glob. Health.

[44] Abe M., Mitani A., Hoshi K., Yanagimoto S. (2020). Large Gender Gap in Oral Hygiene Behavior and Its Impact on Gingival Health in Late Adolescence. Int. J. Environ. Res. Public Health.

[45] Olczak-Kowalczyk D., Gozdowski D., Kaczmarek U. (2019). Oral Health in Polish Fifteen-year-old Adolescents. Oral Health Prev. Dent..

[46] Almas K., Al-Hawish A., Al-Khamis W. (2003). Oral Hygiene Practices, Smoking Habit, and Self-Perceived Oral Malodor among Dental Students. J. Contemp. Dent. Pract..

[47] Eurostat (2017). The Lives of Women and Men in Europe—A Statistical Portrait.

[48] Pienkohs S.P., Meisgeier A., Herrmann J., Graf L., Reichert C.S., Trento G., Neff A. (2023). Factors Affecting the Duration of Surgery in the Management of Condylar Head Fractures. J. Clin. Med..

[49] Damrongsirirat N., Kaboosaya B., Siriwatana K., Subbalekha K., Jansisyanont P., Pimkhaokham A. (2022). Complications Related to Orthognathic Surgery: A 10-Year Experience in Oral and Maxillofacial Training Center. J. Craniomaxillofac. Surg..

[50] Chow L.K., Singh B., Chiu W.K., Samman N. (2007). Prevalence of Postoperative Complications after Orthognathic Surgery: A 15-Year Review. J. Oral Maxillofac. Surg..

[51] Yano K., Ikari K., Takatsuki Y., Taniguchi A., Yamanaka H., Momohara S. (2016). Longer Operative Time Is the Risk for Delayed Wound Healing after Forefoot Surgery in Patients with Rheumatoid Arthritis. Mod. Rheumatol..

[52] Cheng H., Clymer J.W., Po-Han Chen B., Sadeghirad B., Ferko N.C., Cameron C.G., Hinoul P. (2018). Prolonged Operative Duration Is Associated with Complications: A Systematic Review and Meta-Analysis. J. Surg. Res..

[53] El Sheikh Y.M., Seleem M.F.M.A., Fawzy H.H. (2023). Compare between intraoral and extraoral approaches of subcondylar mandibular fracture management. Int. J. Health Sci..

[54] Nam S.M., Kim Y.B., Lee S.J., Kim S.M., Huh J.K. (2019). A comparative study of intraoral versus retromandibular approach in the management of subcondylar fracture. BMC Surg..

[55] Kulkarni V., Chowdhury S.K.R., Ghosh S., Rajkumar K. (2024). Incidence of Facial Nerve Injury and Sialocele Formation Following Mandibular Condylar and Sub-Condyle Fracture Fixation. J. Maxillofac. Oral Surg..

[56] Caruso C. (2022). When Recovery Goes Awry, New findings reveal how recovery progresses following inflammation triggered by injury or illness. News Research.

[57] Mank W., Waqas Azhar W., Brown K. (2025). Leukocytosis. StatPearls [Internet].

[58] Foy B.H., Sundt T.M., Carlson J.C.T., Aguirre A.D., Higgins J.M. (2022). Human acute inflammatory recovery is defined by co-regulatory dynamics of white blood cell and platelet populations. Nat Commun..

[59] Julier Z., Park A.J., Briquez P.S., Martino M.M. (2017). Promoting Tissue Regeneration by Modulating the Immune System. Acta Biomater..

[60] Tsirpanlis G. (2005). Inflammation in Atherosclerosis and Other Conditions: A Response to Danger. Kidney Blood Press. Res..

[61] Kiran D.N., Desai R. (2012). Estimation of C-Reactive Protein Associated with Mandibular Fracture. J. Maxillofac. Oral Surg..

[62] Elbromboly Y., Esawy M.A. (2023). Post-Operative C-Reactive Protein and White Blood Cells Changes Pattern Following Spinal Deformity Surgery and Its Clinical Correlation. J. Orthop. Surg. Res..

[63] Horwich T.B., Kalantar-Zadeh K., MacLellan R.W., Fonarow G.C. (2008). Albumin Levels Predict Survival in Patients with Systolic Heart Failure. Am. Heart J..

[64] Saucedo J.M., Marecek G.S., Wanke T.R., Lee J., Stulberg S.D., Puri L. (2014). Understanding Readmission after Primary Total Hip and Knee Arthroplasty: Who’s at Risk?. J. Arthroplast..

[65] Qin P., Wang Z., Liu L., Xiong Q., Liu D., Min S., Wei K. (2025). The association between BMI and Postoperative Pulmonary Complications in Adults Undergoing Non-Cardiac, Non-Obstetric Surgery: A Retrospective Cohort Study. Assoc. Anaest..

[66] Heo Y.H., Yagi S., Toriyama K., Urken M.L., Nabili V., Kiyokawa K., Takushima A. (2016). Relationship between BMI and postoperative complications with free flap in anterolateral craniofacial reconstruction. Plast. Reconstr. Surg. Glob. Open.

[67] Ebenezer V., Ramalingam B. (2011). Comparison of Approaches for the Rigid Fixation of Sub-condylar Fractures. J. Maxillofac. Oral Surg..

[68] Khoo M.J.W., Ooi A.S.H. (2021). Management of postreconstructive head and neck salivary fistulae: A review of current practices. J. Plast. Reconstr. Aesthet. Surg..

[69] Barrera J.E. (2023). Parotid Duct Injures. Otolaryngol. Facial Plast. Surg..

[70] Lim J.C., Choi E.C. (2008). Treatment of an Acute Salivary Fistula after Parotid Surgery: Botulinum Toxin Type A Injection as Primary Treatment. Eur. Arch. Otorhinolaryngol..

[71] Cohen J., Reed W., Foster M.W., Kahmke R.R., Rocke D.J., Puscas L., Cannon T.Y., Lee W.T. (2022). Octreotide May Improve Pharyngocutaneous Fistula Healing through Downregulation of Cystatins: A Pilot Study. Laryngoscope Investig. Otolaryngol..

[72] Mir A., Guys N., Arianpour K., Svider P.F., Rayess H., Zuliani G., Raza S.N., Lin H. (2019). Negative Pressure Wound Therapy in the Head and Neck: An Evidence-Based Approach. Laryngoscope.

[73] Yang Y.H., Jeng S.F., Hsieh C.H., Feng G.M., Chen C.C. (2013). Vacuum-Assisted Closure for Complicated Wounds in Head and Neck Region after Reconstruction. J. Plast. Reconstr. Aesthet. Surg..

[74] Kaehn K., Eberlein T. (2009). In-vitro test for comparing the efficacy of wound rinsing solutions. Br. J. Nurs..

[75] Wilkins R.G., Unverdorben M. (2013). Wound cleaning and wound healing: A concise review. Adv. Skin Wound Care.

[76] Alvarez-Marin R., Aires-de-Sousa M., Nordmann P., Kieffer N., Poirel L. (2017). Antimicrobial activity of octenidine against multidrug-resistant Gram-negative pathogens. Eur. J. Clin. Microbiol. Infect. Dis..

[77] Assadian O. (2016). Octenidine dihydrochloride: Chemical characteristics and antimicrobial properties. J. Wound Care.

[78] Krasowski G., Junka A., Paleczny J., Czajkowska J., Makomaska-Szaroszyk E., Chodaczek G., Majkowski M., Migdał P., Fijałkowski K., Kowalska-Krochmal B. (2021). In Vitro Evaluation of Polihexanide, Octenidine and NaClO/HClO-Based Antiseptics against Biofilm Formed by Wound Pathogens. Membranes.

[79] Dettenkofer M., Wilson C., Gratwohl A., Schmoor C., Bertz H., Frei R., Heim D., Luft C., Schultz S., Widmer A.F. (2010). Skin disinfection with octenidine dihydrochloride for central venous catheter site care: A double-blind, randomized, controlled trial. Clin. Microbiol. Infect..

[80] Krishna B.V., Gibb A.P. (2010). Use of octenidine dihydrochloride in meticillin-resistant *Staphylococcus aureus* decolonisation regimens: A literature review. J. Hosp. Infect..

[81] Jain J., Arora S., Rajwade J.M., Omray P., Khandelwal S., Paknikar K.M. (2009). Silver nanoparticles in therapeutics: Development of an antimicrobial gel formulation for topical use. Mol. Pharm..

[82] Allawadhi P., Singh V., Khurana A., Khurana I., Allwadhi S., Kumar P., Banothu A.K., Thalugula S., Barani P.J., Naik R.R. (2021). Silver nanoparticle based multifunctional approach for combating COVID-19. Sens. Int..

[83] Lansdown A.B., Sampson B., Laupattarakasem P., Vuttivirojana A. (1997). Silver aids healing in the sterile skin wound: Experimental studies in the laboratory rat. Br. J. Dermatol..

[84] Lansdown A.B. (2006). Silver in health care: Antimicrobial effects and safety in use. Curr. Probl. Dermatol..

[85] Severing A.L., Rembe J.D., Koester V., Stuermer E.K. (2019). Safety and efficacy profiles of different commercial sodium hypochlorite/hypochlorous acid solutions (NaClO/HClO): Antimicrobial efficacy, cytotoxic impact and physicochemical parameters in vitro. J. Antimicrob. Chemother..

[86] Souza M.A., Steier L., Vanin G.N., Brondi M.R., de Melo Carvalho F., de Oliveira Lima C.S., Pinto L.D.R., Rodrigues L.K.A., Colombo A.P.V., de Almeida R.R. (2024). Antimicrobial action, cytotoxicity and erosive potential of hypochlorous acid obtained from an electrolytic device compared with sodium hypochlorite. Clin. Oral Investig..

[87] Farber N.M., Perez-Lloret S., Gamzu E.R. (2015). Design and development of a novel supportive care product for the treatment of sialorrhea in Parkinson’s disease. Curr. Top. Med. Chem..

[88] Naicker P., Anoopkumar-Dukie S., Grant G.D., Kavanagh J.J. (2017). Anticholinergic activity in the nervous system: Consequences for visuomotor function. Physiol. Behav..

[89] Gudis D.A., Soler Z.M. (2021). Nasal Cauterization with Silver Nitrate for Recurrent Epistaxis. N. Engl. J. Med..

[90] Linneman P.K., Litt J. (2022). Hypertrophic Granulation Wounds Treated With Silver Nitrate Sticks or With Topical Steroid: Rate of Wound Closure. J. Burn Care Res..

[91] Martinez J.D., Cardenas J.A., Soria M., Saenz L.M., Estrada K., Delgado S.M., Ionescu M.-A., Busila C., Tatu A.L. (2023). Role of Silver Nitrate Spray for Skin Wound Care in Patients with Toxic Epidermal Necrolysis: Our Experience in 4 Patients. Life.

[92] Hah J.H., Kim B.J., Sung M.W., Kim K.H. (2008). Chemocauterization of congenital fistula from the accessory parotid gland. Clin. Exp. Otorhinolaryngol..

[93] Blythe J.N., Koraitim M., Arcuri F., Brennan P.A. (2016). Novel approach in the treatment of a persistent iatrogenic parotid fistula using AlloDerm^®^—An allogenic acellular dermal matrix. Br. J. Oral Maxillofac. Surg..

